# Neutrophil Gelatinase Associated Lipocalin (NGAL) in Leptospirosis Acute Kidney Injury: A Multicenter Study in Thailand

**DOI:** 10.1371/journal.pone.0143367

**Published:** 2015-12-02

**Authors:** Nattachai Srisawat, Kearkiat Praditpornsilpa, Kanitha Patarakul, Malee Techapornrung, Tinnapop Daraswang, Theerapon Sukmark, Kamol Khositrangsikun, Apinya Fakthongyoo, Petchdee Oranrigsupak, Laksamon Praderm, Ummarit Suwattanasilpa, Sadudee Peerapornratana, Passisd Loahaveeravat, Nattachai Suwachittanont, Thaksa-on Wirotwan, Chayanat Phonork, Sarinya Kumpunya, Khajohn Tiranathanagul, Chintana Chirathaworn, Somchai Eiam-ong, Kriang Tungsanga, Visith Sitprija, John A. Kellum, Natavudh Townamchai, Thai Lepto-AKI study group

**Affiliations:** 1 Division of Nephrology, Department of Medicine, Faculty of Medicine, Chulalongkorn University, and King Chulalongkorn Memorial Hospital, Bangkok, Thailand; 2 Center for Critical Care Nephrology, The CRISMA Center, Department of Critical Care Medicine, University of Pittsburgh School of Medicine, Pittsburgh, Pennsylvania, United States of America; 3 Department of Microbiology and Immunology, Faculty of Medicine, Chulalongkorn University, Bangkok, Thailand; 4 Prapokklao hospital, Chantaburi, Thailand; 5 Krasang hospital, Buriram, Thailand; 6 Tungsong hospital, Nakhon Si Thammarat, Thailand; 7 Maharaj Nakhon Si Thammarat hospital, Nakhon Si Thammarat, Thailand; 8 Uttaradit hospital, Uttaradit, Thailand; 9 Nan hospital, Nan, Thailand; 10 Roiet hospital, Roiet, Thailand; 11 Mahasarakarm hospital, Mahasarakarm, Thailand; 12 Queen Saovabha Memorial Institute, Thai Red Cross, Bangkok, Thailand; University of São Paulo School of Medicine, BRAZIL

## Abstract

AKI is one of the most serious complications of leptospirosis, an important zoonosis in the tropics. Recently, NGAL, one of the novel AKI biomarkers, is extensively studied in various specific settings such as sepsis, cardiac surgery, and radiocontrast nephropathy. In this multicenter study, we aimed to study the role of NGAL as an early marker and an outcome predictor of leptospirosis associated AKI. Patients who presented with clinical suspiciousness of leptospirosis were prospectively enrolled in 9 centers from August 2012 to November 2014. The first day of enrollment was the first day of clinical suspicious leptospirosis. Blood and urine samples were serially collected on the first three days and day 7 after enrollment. We used three standard techniques (microscopic agglutination test, direct culture, and PCR technique) to confirm the diagnosis of leptospirosis. KDIGO criteria were used for AKI diagnosis. Recovery was defined as alive and not requiring dialysis during hospitalization or maintaining maximum KDIGO stage at hospital discharge. Of the 221 recruited cases, 113 cases were leptospirosis confirmed cases. Thirty seven percent developed AKI. Median uNGAL and pNGAL levels in those developing AKI were significantly higher than in patients not developing AKI [253.8 (631.4) vs 24.1 (49.6) ng/ml, p < 0.001] and [1,030 (802.5) vs 192.0 (209.0) ng/ml, p < 0.001], respectively. uNGAL and pNGAL levels associated with AKI had AUC-ROC of 0.91, and 0.92, respectively. Both of urine NGAL and pNGAL level between AKI-recovery group and AKI-non recovery were comparable. From this multicenter study, uNGAL and pNGAL provided the promising result to be a marker for leptospirosis associated AKI. However, both of them did not show the potential role to be the predictor of renal recovery in this specific setting.

## Introduction

Leptospirosis is an important zoonosis especially in the tropics. However, with the impact of world globalization, there are also reports of this disease as sporadic cases in developed countries. A recent report has shown the annual incidence of leptospirosis in Thailand was 48.9 per million population, thus ranking 7^th^ in the world in terms of incidence [1].

Acute Kidney Injury (AKI) is one of the most serious complication of leptospirosis. The incidence of AKI in leptospirosis by using the RIFLE AKI criteria was up to 84% [2]. This is higher than the average AKI incidence in the Intensive Care Unit (ICU) [3] and more than twice as high as seen in patients with community acquired pneumonia in the US [4]. In this specific setting the kidney is injured by direct effects (direct invasion of the organism) and by indirect effects such as dehydration, rhabdomyolysis, and hemorrhagic shock [5]. One of the most robust biomarkers for AKI is neutrophil gelatinase associated lipocalin (NGAL). Previous studies have used NGAL as an early marker of AKI [6] and as an outcome predictor [7,8]. However, only one study explored the role of NGAL in leptospirosis patients [9]. Benefits from biomarkers to predict AKI in leptospirosis would include early triage patients from primary hospital to tertiary care facilities, early treatment interventions such as fluid resuscitation, optimizing tissue oxygenation and perfusion possibly preventing AKI progression, and finally, early discontinuation of nephrotoxic drugs.

We conducted a prospective observational study to measure NGAL in urine and plasma of hospitalized patients suspected of having leptospirosis. Using the KDIGO criteria to classify AKI status [10], first, we analyzed baseline characteristics between AKI patients and non AKI patients. Second, we compared the differences in urine and plasma NGAL (uNGAL, pNGAL) concentration by AKI and renal recovery status. Third, we examined whether uNGAL and pNGAL level associated with AKI and predicted renal non-recovery in a multivariable model adjusting for clinical parameters.

## Materials and Methods

### Ethics statement

Study protocol was approved by The Institutional Review Board of Faculty of Medicine, Chulalongkorn University, and The Institutional Review Board of Ministry of Public Health of Thailand. All participants accepted the protocol and provided written informed consent.

### Patients and study design

This study was a multicenter, prospective, cohort study of patients presenting with clinical suspicion of leptospirosis. All the cases were hospitalized. Specific inclusion criteria included high fever (BT higher than 38°c), severe myalgia, and history of exposure to reservoir animals. The patients need to be first admitted into the participating centers, not referring from the non-participating centers. We excluded the patients who suffered from known other infectious disease. This study was conducted in 9 centers in 8 provinces around Thailand during August 2012 to November 2014. Of the nine participating hospitals, 2 centers were from the Northern part, 3 centers from the Northeastern part, 1 center from the Eastern part and the other three centers from the Southern part. For the level of care, 2 centers were the referral/tertiary care hospital, 7 centers were the provincial hospital. Blood and urine were serially collected on the first three days after study enrollment and on day 7.

#### Sample collection and Biomarkers assay

A 12-ml blood sample was taken and well mixed on the day 1, day 2, day 3 and day 7 after enrollment. The first day of enrollment was the first day of clinical suspicious leptospirosis. A 30-ml urine sample was obtained on the same day. Urine samples were poured into 50-ml conical centrifuge tubes. Both of plasma and urine were centrifuged for 10 minutes at 1000 G at 4°C, and frozen at -20°C until shipped to the central laboratory. Samples were then stored at -80°C until analyzed. uNGAL was measured by ELISA (R&D, Minneapolis, MN, USA), following the manufacturers’ instructions. Plasma NGAL was tested using the Triage NGAL kit (Alere, San Diego, CA, USA). Both biomarkers were tested on the first day of enrollment. All samples were analyzed in duplicate.

#### Definitions

We used three standard techniques; microscopic agglutination test (MAT), direct culture, and PCR technique, to confirm leptospirosis. In brief, MAT was performed by using the standard protocol of the World Health Organization (WHO) guideline [11]. A positive MAT was defined as a single serum titer of >1:100 or a 4-fold rise in pair serum. We used the single serum cut point of > 1:100 based on previous study [12]. For direct culture of leptospires, one drop of whole blood was cultured into 4 mL liquid Ellinghausen–McCullough–Johnson–Harris (EMJH) at 29°C for 2 weeks. Detection for leptospires was accomplished by direct observation using Dark-field microscopy. For PCR technique, DNA was extracted from urine samples using a High Pure PCR Template Preparation kit (Roche Diagnostics, Germany). The two primers used for amplification of LipL 32 gene were as follows 45F primers (5’ AAG CAT TAC CGC TTG TGG TG3’) and 287R primers (5’ CGA ACT CCC ATT TCA GCG AT 3’), PCR reactions of urine samples were performed in a final volume of 20 μl, corresponding to 2 μl of genomic DNA and 18 μl of reaction mix containing 25 mM of each dNTP; 0.1 μl of Taq DNA polymerase; 0.4 μl of each primer in 25 mM MgCl2 and 10x KCl under 13.5 μl DW. The PCR program consisted of an initial cycle of 94°C for 10 min, followed by 40 cycles of 94°C for 1 min, 55°C for 1 min and 72°C for 1 min and a final extension step at 72°C for 7 min. PCR products were run on a 1% agarose gel with ethidium bromide and photographed.

We used the term ***“All cases”*** for patients who were clinical suspicious of leptospirosis and were enrolled into the cohort. ***“Leptospirosis cases”*** were defined if any one of the above tests were positive.

We used KDIGO guideline to classify AKI status [10]. Due to the limitation of urine output data, we use only serum creatinine criteria. Definitions of the baseline serum creatinine was the lowest value between the a. the first creatinine recorded on the day of hospital admission or 2) the back calculation from the Modification on Diet in Renal Disease (MDRD) equation for equation for serum creatinine using a GFR of 75mL/min/1.73m^2^ [13].

Recovery was defined as alive and not requiring dialysis during hospitalization and not having a persistent KDIGO stage at hospital discharge (i.e. patients had to improve by at least one KDIGO stage to be considered as recovery) [7,13].

### Statistical analyses

Clinical characteristics and biomarkers on the day of enrollment were compared between patients with AKI and those without AKI and between renal recovery patients and non-recovery patients at hospital discharge time. Categorical data were expressed as proportions and compared using a Chi-square test. Continuous data were expressed as mean ± standard deviation and compared using the student’s t-test or were expressed as median ± intra-quartile range (IQR) and compared using Mann-Whitney U test, as appropriate. We fitted an inclusive logistic regression model using biomarkers to predict the probability of AKI and recovery from AKI, and expressed these results as odds ratios (OR), and 95% confidence intervals (CI). To assess the predictive ability of single and multiple biomarkers, receiver operating characteristic (ROC) curves were generated and the area under the curve (AUC) was calculated. Statistical analyses were performed using SPSS version 17.0 and GraphPad PRISM version 5.1. P values < 0.05 were considered to indicate statistical significance.

## Results

### Patients clinical characteristics

**Fig 1:**
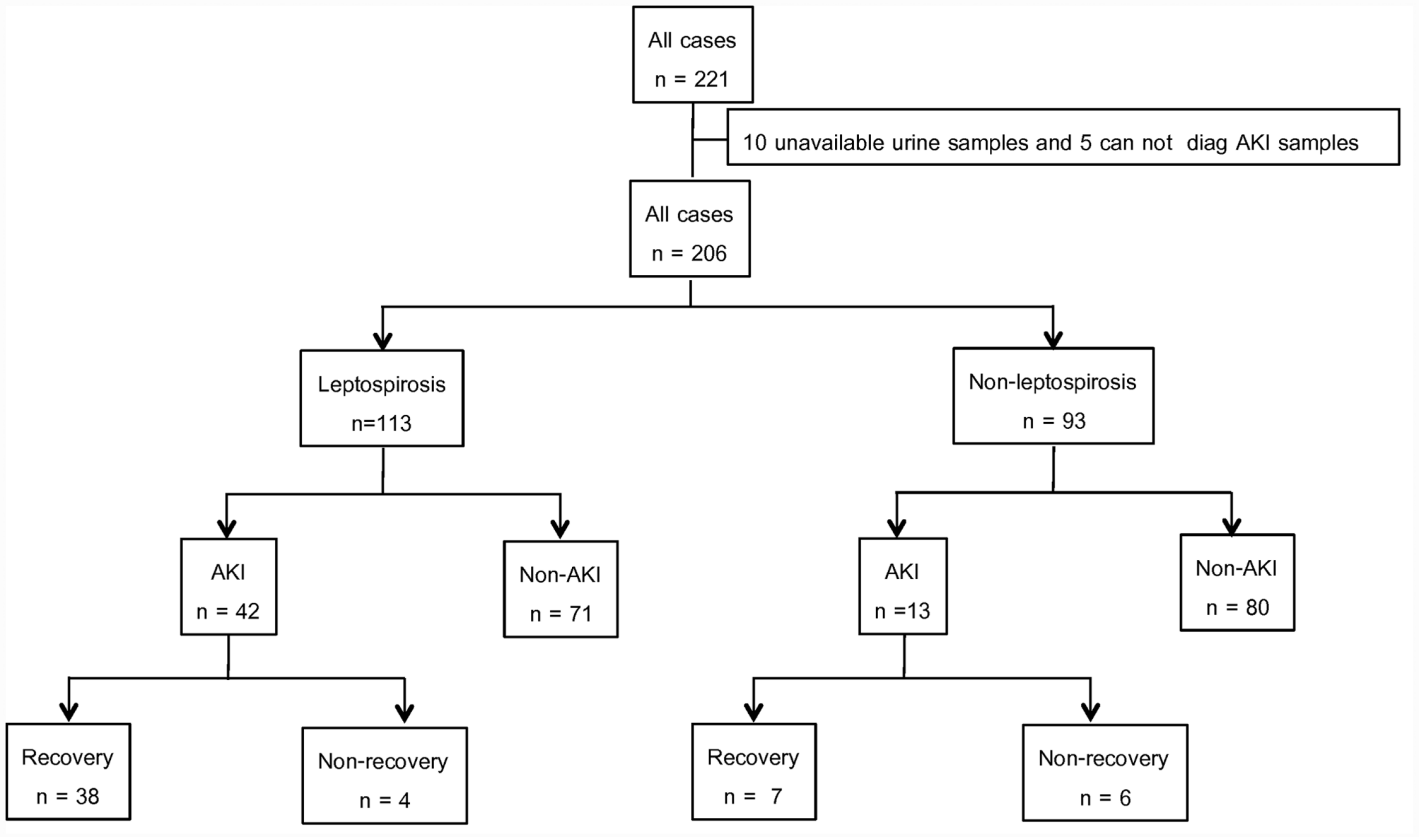
Subject disposition for the lepto Thai study cohort.

Of the 221 subjects with clinical suspicion of leptospirosis (all cases), urine samples were unavailable in 10. These cases and 5 additional patients that could not be identified for AKI status were excluded (Fig 1). Of the remaining 206, 55 (26%) were diagnosed with AKI. Of the 55 cases of AKI, 7.3% had KDIGO stage1, 16.4% had KDIGO stage2, and 76.4% had KDIGO stage3. AKI patients had the recovery rate at 81.8% (Fig 1). Twenty nine percent of AKI patients required dialysis support. AKI patients had mortality rate 10.1% while non-AKI patients had only 0.6%. AKI patients had lower body temperature, more leukocytosis, lower hemoglobin, more thrombocytopenia, higher bilirubin (TB/DB), higher SGOT/SGPT, and lower sodium than non-AKI patients (Table 1).

**Table 1:** Patient characteristics by AKI status on the first day of enrollment into the study.

Characteristic	All cases (n = 206)			Leptospirosis cases (n = 113, 54.9%)			Non-leptospirosis cases (n = 93, 45.1%)		
	AKI (n = 55, 26.7%)	Non-AKI (n = 151)	P-value	AKI (n = 42, 37.1%)	Non-AKI (n = 71)	p-value	AKI (n = 13, 14.0%)	Non-AKI (n = 80)	p-value
Gender, Male (%)	44(81.5)	119(78.3)	0.70	35(85.4)	59(81.9)	0.80	9(69.2)	60(75.0)	0.74
Fever day, days	4.5(2.1)	3.9(2.4)	0.08	4.7(2.2)	3.8(2.6)	0.21	3.6(1.6)	3.94(2.3)	0.65
Age, years	46.3(17.1)	41.6(14.8)	0.06	43.0(12.9)	36.6(11.1)	0.17	46.1(22.6)	35.3(16.2)	0.28
Body temperature, °c	37.1(2.2)	38.4(1.1)	0.001	37.4(1.1)	38.0(1.1)	0.07	36.3(4.0)	38.78(1.1)	0.003
SBP, mmHg	107.8(21.2)	111.7(20.0)	0.30	109.1(22.9)	110.2(18.6)	0.89	104.1(16.2)	114.9(21.9)	0.06
DBP, mmHg	64.6(15.0)	68.9(12.9)	0.06	65.4(15.2)	68.8(13.2)	0.35	62.3(14.9)	69.5(13.1)	0.039
Creatinine, mg/dL	5.0(1.9)	1.0(0.3)	0.009	5.1(2.2)	1.0(0.6)	<0.001	4.1(3.5)	0.9(0.4)	<0.001
WBC x 10^3^/uL	12.7(10.6)	7.9(4.9)	<0.001	13.3(9.8)	7.9(8.1)	0.002	9.3(10.6)	7.7(5.1)	0.10
Hgb, g/dL	11.3(2.9)	12.4(3.4)	<0.001	11.0(1.9)	11.6(2.8)	0.019	13.2(3.2)	12.2(2.0)	0.56
Platelet x 10^3^/uL	88.6(85.5)	166.8(114.0)	0.001	39.0(91.0)	150.5(90.3)	<0.001	68.5(109.0)	139.0(117.3)	0.002
TB, mg/dL	4.0(6.9)	1.2(2.9)	<0.001	4.3(13.1)	1.2(3.7)	0.007	3.9(4.4)	1.3(1.5)	0.04
DB, mg/dL	2.3(4.9)	0.3(1.3)	<0.001	2.3(5.5)	0.3(1.4)	<0.001	2.0(3.1)	0.5(0.7)	0.007
SGOT, u/L	61(124)	49(71)	<0.001	58(75)	49(81)	0.037	544(2770)	47(85)	0.03
SGPT, u/L	64(87)	46(70)	0.009	61(80)	46(80)	0.05	209(5393)	44(83)	0.09
Na, mEq/L	133.0(8)	135.0(7)	0.023	134.8(4.4)	134.5(4.1)	0.61	134.5(5.8)	128.6(28.7)	0.51
K, mEq/L	4.0(1.1)	3.5(0.5)	0.56	3.8(0.5)	3.6(0.6)	0.28	4.6(1.9)	3.36(0.5)	0.09
HCO_3_^−^, mEq/L	19.0(4.4)	22.1(7.0)	0.06	19.8(4.3)	22.0(7.5)	0.28	16.5(4.0)	21.9(6.6)	0.001
RRT (%)	16(29)	0		10(24)	0		6(46)	0	

SBP: systolic blood pressure, DBP: diastolic blood pressure, WBC: white blood cell, Hgb: hemoglobin, TB: total bilirubin, DB: direct bilirubin, SGOT: Serum Glutamic Oxaloacetic Transaminase, SGPT: Serum Glutamic, Pyruvic Transaminase, HCO_3_^−^: bicarbonate

Values in the table is mean (s.d.) for Fever day, Age, Body temperature, SBP, DBP, Hgb, Na, K, HCO3; median (IQR) for Creatinine, WBC, Platelet, TB, DB, SGOT, SGPT and count (%) for Gender, and RRT, RRT: renal replacement therapy.

The diagnosis of leptospirosis was confirmed in 113 patients (54.9%), and about one third (37.2%) developed AKI. Of the 42 AKI cases, most of them had KDIGO stage 3 (33 cases, 78.6%), followed by KDIGO stage 2 (7 cases, 16.7%), and KDIGO stage 1 (2 cases. 4.7%). Twenty four percent of AKI patients need dialysis support. The mortality rate in AKI patients was 9.5% and no one in non-AKI patients was died. Again, Leptospirosis patients who developed AKI were likely to have more leukocytosis, more thrombocytopenia, lower hemoglobin, more thrombocytopenia, higher bilirubin level, and higher serum glutamic oxaloacetic transaminase (SGOT) and serum glutamic pyruvic transaminase (SGPT) than non-AKI patients (Table 1).

By contrast, only 13 from the 93 (14.0%) non-leptospirosis patients, developed AKI (Fig 1). Of the 13 AKI cases, 15.4% had KDIGO stage1 and KDIGO stage 2. While 69.2% had KDIGO stage 3. Forty six percent of AKI patients required dialysis support. AKI patient had higher mortality rate than non-AKI patients, 15.4% vs 1.2%. Patients with AKI had lower diastolic blood pressure, more thrombocytopenia, higher bilirubin (TB/DB), and higher SGOT than non-AKI patients (Table 1).

### Biomarker concentrations by AKI and recovery status

**Fig 2:**
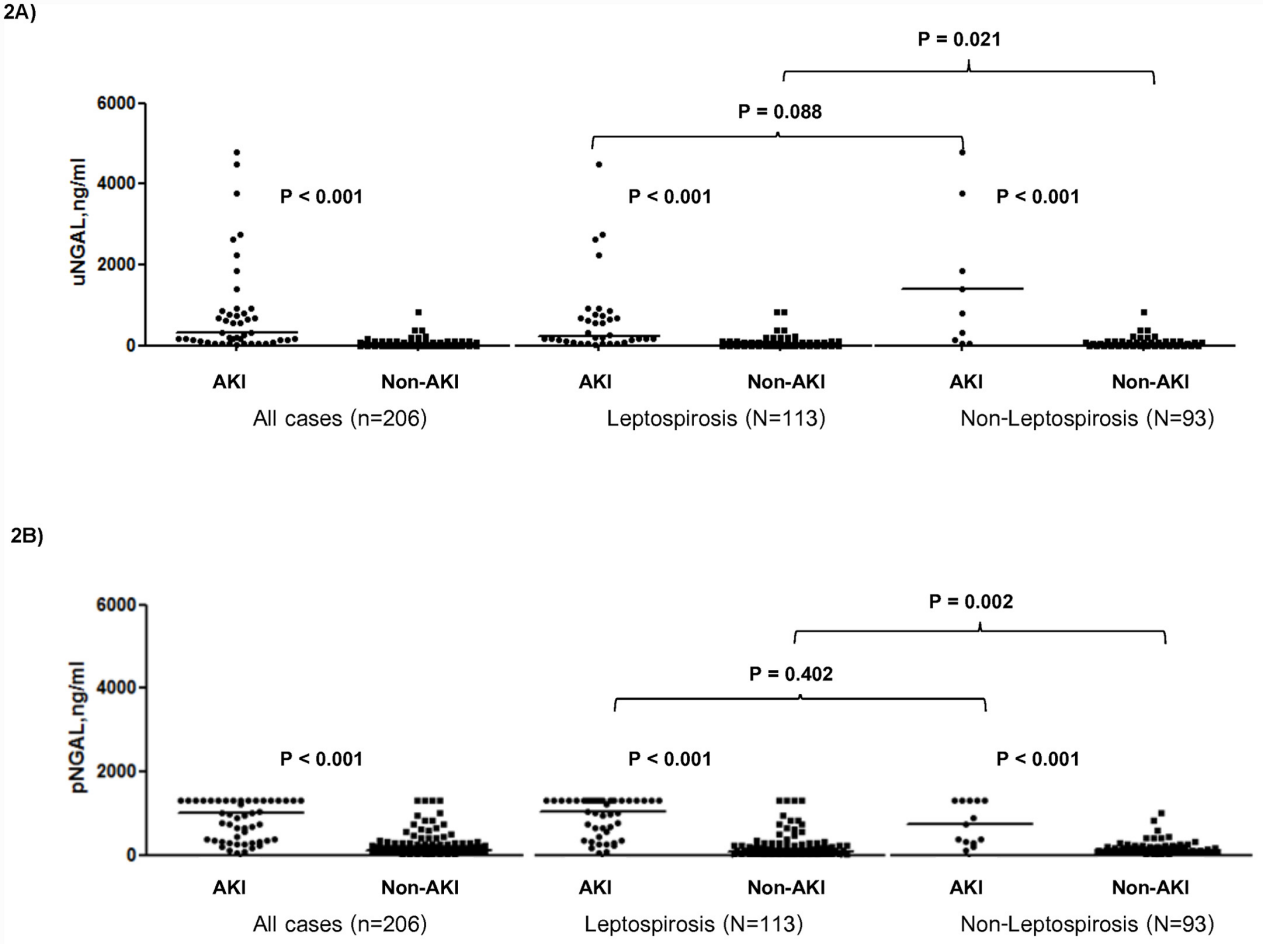
Urine and plasma neutrophil gelatinase-associated lipocalin (NGAL) concentration stratified by AKI status.

**Fig 3:**
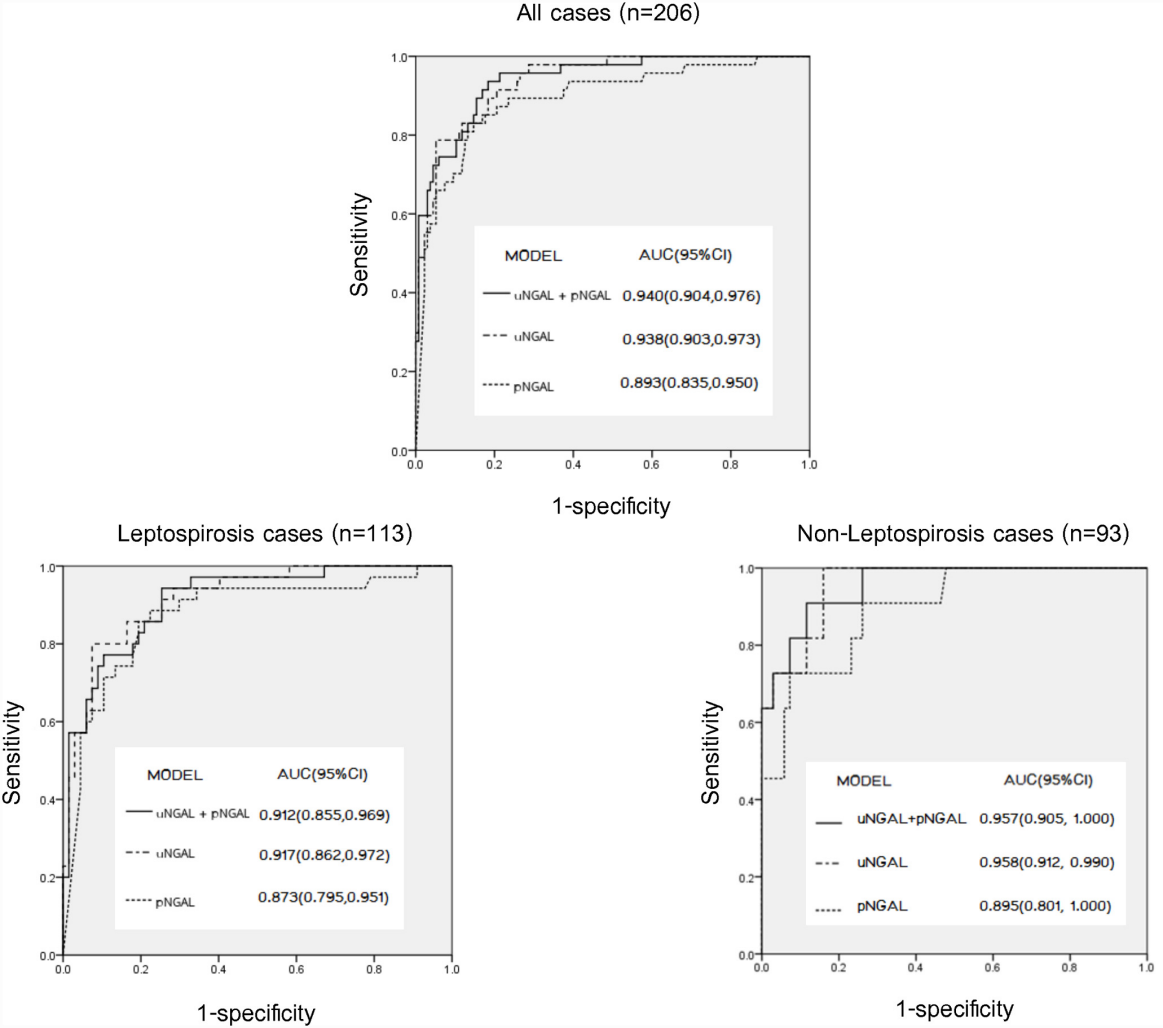
The area under the curve (AUC) for the association between biomarkers and AKI: pNGAL alone, uNGAL alone, and combined model.

First, focusing on all patients with suspected Leptospirosis, AKI patients had significantly higher urine and plasma NGAL (uNGAL, pNGAL) than non-AKI patients on the 1^st^ day of enrollment, uNGAL; AKI vs non-AKI 435.5 ng/ml (interquartile range (IQR) 918.6) vs 18.3 ng/ml (IQR 39.9), P < 0.001, pNGAL: AKI vs non-AKI 1,015 ng/ml (IQR 919.8) vs 124.0 ng/ml (IQR 166.0), P < 0.001 (Fig 2A and 2B). However, neither uNGAL nor pNGAL were significantly different between patients recovering from versus those not recovery: uNGAL recovery vs non-recovery 435.5 ng/ml (IQR 904.0) vs 501.4 (IQR 1499.9) ng/ml, P = 0.78; pNGAL recovery vs non-recovery 993.0 ng/ml (IQR 895.0) vs 1160.0 ng/ml (IQR 1056.5), P = 0.91 (Fig 3A and 3B).

When we restricted the analysis to patients without evidence of AKI (i.e. normal creatinine) on enrollment (4 of 55 cases), uNGAL was still significantly higher than in patients not developing AKI: 1199.9 ng/ml (IQR 3141.75.) vs 19.52 ng/ml (IQR 39.94), P = 0.005. However the level of pNGAL between these two group was comparable: 385.5 ng/ml (IQR 628.0) vs 161 ng/ml (IQR 169.8), P = 0.68.

For patients with confirmed leptospirosis, median uNGAL and pNGAL on the 1^st^ day of enrollment were also significantly higher for patients with AKI: uNGAL, AKI vs non-AKI 253.8 ng/ml (IQR 631.4) vs 24.1 ng/ml (IQR 49.6), P < 0.001; pNGAL, AKI vs non-AKI 1,030 (IQR 802.5) vs 192.0 (IQR 209.0), P < 0.001 (Fig 2A and 2B). However, again patients recovering from AKI did not demonstrate differences in median uNGAL or pNGAL concentrations compared to those not recovering: recovery vs non-recovery, uNGAL, 253.8 ng/ml (IQR 615.4) vs 531.8 ng/ml (IQR 1754.3), P = 0.65; pNGAL, 1030.0 (IQR 802.5) vs 192.0 (IQR 209.0), P = 0.93.

For patients without leptospirosis, we also demonstrated the higher median uNGAL and pNGAL concentrations among those with AKI: uNGAL (140.3 ng/ml (IQR 4667.7) compared with those without (15.1 ng/ml (IQR 21.0), P < 0.001, pNGAL: AKI vs non-AKI 725.0 ng/ml (IQR 994.0) vs 97.5 ng/ml (IQR 137.0), P < 0.001 (Fig 2A and 2B). Neither biomarker discriminated between recovery and non-recovery.

When comparing between leptospirosis AKI and non-leptospirosis AKI, the median uNGAL and pNGAL of leptospirosis AKI and non-leptospirosis AKI were comparable, P = 0.09 and P = 0.40, respectively (Fig 2A and 2B).

We have also stratified the level of biomarkers by day of fever (first 3 days, and after day 3 of fever) to decrease the lead time bias. For all cases, we significantly found uNGAL and pNGAL level of AKI group higher than non-AKI group on the first three day of fever (P < 0.001, P < 0.001, respectively). From day 4 after onset of fever, both uNGAL and pNGAL still showed the higher level in AKI group than in non-AKI group (P < 0.001, P < 0.001, respectively) (Table 2A). Neither uNGAL nor pNGAL exhibited differences between recovery and non-recovery groups at any study period. For the leptospirosis confirmed patients and non-leptospirosis patients, we also found found uNGAL and pNGAL level of AKI group higher than non-AKI group on the first three day of fever and after day 3 of fever (Table 2B and 2C). Again, we did not found significant difference of uNGAL or pNGAL exhibited between recovery and non-recovery groups at any study period.

**Table 2:** Analysis of biomarkers stratified by day of fever between AKI and non-AKI (A) in all cases, (B) in leptospirosis cases, and (C) in non-leptospirosis cases.

Biomarkers	Day of fever	AKI	Non-AKI	P-value
**A. In all cases (n = 206)**				
uNGAL, ng/mL	day 1 –day 3	179.4(4733.86)	16.1(35.94)	<0.001
	day 4 up	561.99(688.43)	21(48.02)	<0.001
pNGAL, ng/mL	day 1 –day 3	891.65(682)	102(131.5)	<0.001
	day 4 up	1300(644)	173(221.5)	<0.001
Serum creatinine,,mg/dL	day 1 –day 3	3.6(2.95)	0.96(0.32)	<0.001
	day 4 up	4.54(2.86)	0.88(0.38)	<0.001
**B. In leptospirosis cases (n = 113)**				
uNGAL, ng/mL	day 1 –day 3	158.4(867.7)	20.8(45.7)	<0.001
	day 4 up	550.3(583.3)	23.2(64.4)	<0.001
pNGAL, ng/mL	day 1 –day 3	945(732)	111(134)	0.001
	day 4 up	1300(593.5)	211.5(147.3)	<0.001
Serum creatinine, mg/dL	day 1 –day 3	3.6(2.95)	0.98(0.32)	<0.001
	day 4 up	5.1(2.5)	0.88(0.45)	<0.001
C. In non-leptospirosis cases (n = 93)				
uNGAL, ng/mL	day 1 –day 3	4793.2(7941.5)	13.3(20.6)	0.001
	day 4 up	1403.1(2355.6)	16.8(17.7)	<0.001
pNGAL, ng/mL	day 1 –day 3	725(818.8)	97.5(134.25)	0.001
	day 4 up	391(1112)	100(133.25)	0.002
Serum creatinine, mg/dL	day 1 –day 3	2.36(3.68)	0.91(0.36)	<0.001
	day 4 up	4.22(5.28)	0.88(0.28)	<0.001

### Biomarkers for diagnosis of AKI and prediction renal recovery

Fig 3A illustrated the ROC curves for uNGAL and pNGAL. For diagnosis AKI in all cases (leptospirosis suspected cases) the area under the ROC curve (AUC) was 0.94 (95% confidence interval (CI) 0.90–0.97) for uNGAL and 0.89 (95% 0.84–0.95) for pNGAL. The AUCs for uNGAL and pNGAL for prediction of renal non-recovery in all cases were 0.53 (95% CI 0.33–0.74) and 0.49 (95% CI 0.23–0.76), respectively. For diagnosis AKI, we chose the cut-off which provided the maximum summation of sensitivity and specificity. The sensitivity and specificity together were maximized at the 78.2 ng/ml cut-off for uNGAL and at the 296.5 ng/ml cut-off for pNGAL (Table 3A). Focusing on the predicting model for AKI and renal recovery in all cases, we found uNGAL and pNGAL independently associated with AKI, odds ratio (OR) 18.1 (95% CI 2.0–160.5), P = 0.009, and OR 1.59 (1.28–1.97), P < 0.001, respectively) (Table 4A). Neither uNGAL nor pNGAL were predictive of renal non-recovery in the adjusted model. We ran a stepwise analysis combining uNGAL and pNGAL in order to assess the discriminatory effects of these biomarkers in association with AKI and predicting renal recovery. The model comprising two biomarkers (Fig 3A) didn’t increase the association between NGAL and AKI with AUC of 0.94 (0.90–0.98). Again, the combined biomarker model didn’t improve the prediction of renal non-recovery with AUC of 0.55 (95% CI 0.31–0.79). To improve the prediction of renal outcome, we have combined the clinical parameters including body temperature and systolic blood pressure with the uNGAL and pNGAL. We found the improvement of prediction with the AUC of 0.80 (0.44,1.00) (S1 Table).

**Table 3:** Urine NGAL, plasma NGAL concentration at the best cut-off values for diagnosis AKI (A) in all cases, (B) in leptospirosis cases, and (C) in non-leptospirosis cases.

Biomarkers	cutoff, ng/mL	Sensitivity	Specificity	PPV	NPV
**A. In all cases (n = 206)**					
uNGAL	78.2	0.848	0.884	0.709	0.946
pNGAL	296.5	0.852	0.841	0.657	0.941
**B. In leptospirosis case (n = 113)**					
uNGAL	78.2	0.861	0.851	0.756	0.919
pNGAL	327	0.854	0.814	0.729	0.905
**C. In non-leptospirosis cases (n = 93)**					
uNGAL	44	0.909	0.841	0.476	0.983
pNGAL	185.5	0.846	0.734	0.344	0.967

**Table 4:** Analysis of biomarkers associated with AKI (A) in all cases, (B) in leptospirosis cases, and C) in non-leptospirosis cases.

Biomarkers/Clinical Parameters	Odds ratio (95%CI) unadjusted	P-value	Odds ratio(95%CI) adjusted	P-value
**A. For all patients (n = 206)**				
uNGAL x 100 ng/mL	2.58(1.68,3.95)	<0.001	18.10(2.04,160.52)^a^	0.009
pNGAL x 100 ng/mL	1.48(1.33,1.64)	<0.001	1.59(1.28,1.97) ^b^	<0.001
Serum creatinine x 0.1, mg/dL	1.27(1.17,1.39)	<0.001		
Body temperature, °c	0.67(0.51,0.88)	0.005	0.19(0.06)0.64 ^a^	0.008
WBC x 5000/uL	1.10(1.04,1.16)	0.001		
Hgb, g/dL	0.79(0.69,0.92)	0.002		
Platelet x 10000/uL	0.88(0.84,0.93)	<0.001	0.92(0.84,1.01) ^a^	0.078
			0.90(0.83,0.96) ^b^	0.003
TB, mg/dL	1.08(1.01,1.62)	0.024		
DB, mg/dL	1.15(1.02,1.29)	0.020		
SGOT x 10, u/L	1.05(0.99,1.12)	0.088		
SGPT x 10, u/L	1.01(1.00,1.02)	0.204		
HCO_3_^−^, mEq/L	0.94(0.89,0.99)	0.013	0.90(0.82,0.99) ^b^	0.026
B. For leptospirosis cases (n = 113)				
uNGAL x 100 ng/mL	2.58(1.68,3.95)	<0.001	14.07(1.45,136.80) ^a^	0.023
pNGAL x 100 ng/mL	1.40(1.25,1.58)	<0.001	1.45(1.18,1.78) ^b^	<0.001
Serum creatinine x 0.1, mg/dL	1.20(1.11,1.30)	<0.001		
WBC x 5000/uL	1.12(1.03,1.21)	0.006		
Hgb, g/dL	0.81(0.68,0.97)	0.023		
Platelet x 10000/uL	0.91(0.86.0.96)	0.001	0.81(0.68,0.96) ^a^	0.013
			0.90(0.83,0.97) ^b^	0.008
TB, mg/dL	1.07(1.00,1.16)	0.064		
DB, mg/dL	1.16(1.00,1.34)	0.044		
SGOT x 10, u/L	1.02(0.96,1.08)	0.575		
SGPT x 10, u/L	1.00(0.99,1.02)	0.786		
C. For non-leptospirosis cases (n = 93)				
uNGAL x 100 ng/mL	2.11(0.99,4.48)	0.051	2.11(0.99,4.48)^a^	0.051
pNGAL x 100 ng/mL	1.58(1.25,2.02)	<0.001	2.43(1.18,4.98)^b^	0.015
Serum creatinine x 0.1, mg/dL	3.2(1.01,10.2)	0.049		
Body temperature, °c	0.19(0.42,1.04)	0.073		
DBP	0.98(0.93,1.03)	0.379		
Platelet x 10000/uL	0.9(0.83,0.98)	0.012		
TB, mg/dL	1.02(0.89,1.22)	0.472		
DB, mg/dL	1.02(0.85,1.33)	0.180		
SGOT x 10, u/L	1.02(0.99,1.06)	0.112	1.02(0.99,1.03)^b^	0.157
HCO3-, mEq/L	0.18(0.48,0.81)	<0.001		

WBC: white blood cell, Hgb: hemoglobin, TB: total bilirubin, DB: direct bilirubin, SGOT: serum glutamic oxaloacetic transaminase, SGPT: serum glutamic, pyruvic transaminase, HCO_3_^−^: bicarbonate

^a^ adjusted for uNGAL,

^b^ adjusted for pNGAL.

For the association between NGAL and AKI in leptospirosis cases, the AUC-ROC was 0.91 for uNGAL, and 0.92 for pNGAL (Fig 3B). The best cut-off for diagnosis AKI was 78.2 ng/ml for uNGAL, and 327 ng/ml for pNGAL (Table 3B). In the adjusted model, we found uNGAL and pNGAL independently associated with AKI, OR 14.1, P = 0.023, and OR 1.45, P < 0.001, respectively (Table 4B). Neither uNGAL nor pNGAL were predictive of renal non-recovery in the adjusted model. In a stepwise analysis combining uNGAL and pNGAL in association with AKI, the combining two biomarkers didn’t increase the association with AKI, AUC of 0.91 (Fig 3B).

For the association between NGAL and AKI in non-leptospirosis cases, the AUC-ROC was 0.96 for both uNGAL and pNGAL (Fig 3C). The best cut off for diagnosis AKI was 44.0 ng/ml for uNGAL and 185.5 ng/ml for pNGAL (Table 3C). In the adjusted model, we found only pNGAL independently associated with AKI, OR 2.43, P = 0.015 (Table 4C). Neither uNGAL nor pNGAL were predictive of renal non-recovery in the adjusted model. In a stepwise analysis combining uNGAL and pNGAL in association with AKI, the combining two biomarkers didn’t increase the association of biomarkers and AKI with AUC of 0.96 (Fig 3C).

## Discussion

In this study, we found that both uNGAL and pNGAL concentrations were significantly higher in patients with all cases (suspected leptospirosis), leptospirosis cases, and non-leptospirosis cases, who developed AKI. Both biomarkers had good association with AKI on the first day of presentation. In leptospirosis cases, uNGAL > 78.2 ng/ml and pNGAL > 327.0 ng/ml, associated with AKI by a sensitivity of 86.1% and 85.0% and specificity of 85.1% and 81.4% respectively. Using these cutoffs, the positive (PPV) and negative predictive values (NPV) for uNGAL were 75.6% and 91.9%, and for pNGAL were 72.9% and 90.5%. Moreover, uNGAL and pNGAL remained strong association with AKI by a covariate-adjusted model. Combining two markers together didn’t improve the association with AKI. Neither uNGAL nor pNGAL were the predictors of renal recovery.

To our knowledge, this is the largest study to examine the role of NGAL in AKI and renal recovery for patients with suspected or confirmed leptospirosis. AKI can be a diagnostic challenge in patients with leptospirosis because serum creatinine may be interfered by many factors such as volume overload, rhabdomyolysis, and jaundice. These confounding factors together make serum creatinine less sensitive and specific in the setting of leptospirosis. Most of leptospirosis patients live in rural areas and shortages of nephrologists and dialysis equipment are common. Availability of specific novel biomarkers would accelerate the timing of AKI detection and triage the leptospirosis AKI patients to tertiary care hospitals. During the past few years, uNGAL and pNGAL have been tested as markers for early diagnosis of AKI in various setting: sepsis, ischemic, post-transplantation [14–21]. Associations between NGAL and clinical and laboratory markers of severity such as WBC count, bilirubin level, or lower of platelet count and AKI are not unexpected and corresponded with the previous studies [22,23].

In our cohort, there was no difference in median uNGAL levels between leptospirosis AKI patients and non-leptospirosis AKI patients. Although the optimal cut-off for uNGAL (about 80 ng/ml) to diagnosis leptospirosis AKI in our study seemed to be a bit lower than the other studies (usual cutoff for general septic AKI was 100–150 ng/ml) [24].

The higher sensitivity of uNGAL in diagnosis AKI in leptospirosis could be due to the fact that leptospirosis directly invades distal tubule epithelium. We found that pNGAL was less association with AKI than uNGAL (AUC 0.87 VS 0.92 for leptospirosis cases). This might be explained by plasma NGAL can be produced in various organs such as liver, colon and lung during the process of inflammation/infection [23]. Moreover, inflammatory cell like neutrophils, monocytes, and other immune-competent cells can also synthesized and release NGAL into systemic circulation [25]. The last, declining of filtration rate during AKI may slow down the elimination of systemic NGAL and result in the rising of concentrations of pNGAL [26].

There are several limitations to our study. First, due to the study design and variable timing of hospital admission, we cannot test all patients from the first day of fever. Therefore most of our patients had increased serum creatinine since the first day of enrollment. Therefore, the interpretation of the result for the role of NGAL prediction leptospirosis AKI need to understand this limitation. However, we have stratified patients by day of fever and still found that the NGAL level was significantly higher in patients with AKI than in patients without. Second, we have measured biomarkers at only a single time point (on the first day of enrollment) and could not assess the value of uNGAL and pNGAL as repeated measures. Nevertheless, risk stratification and prognostication using a biomarker is likely to be useful only when biomarker concentrations are measured early. Third, we were unable to measure process of care variables such as the effect of co-interventions on biomarkers and its influence on development of AKI. Fourth, we found that uNGAL and pNGAL could not predict renal non-recovery. Our finding should be interpreted with caution because the limitation number of patients in our study. Only 10 patients from 55 AKI patients did not recover. However, it is notable that neither biomarker could predict recovery when we have observed reasonable performance for this indication in other patient populations including community-acquired pneumonia [7] and acute renal failure receiving renal replacement therapy [8].

Our study has several strengths. First, the study design is a multicenter study testing biomarkers in hospitalized patients with leptospirosis, therefore the results are highly applicable to this specific population. Second, we chose the first day of presentation to medical attention (the first day of clinical suspicious leptospirosis) as the time to test biomarkers. This time point was match to real clinical situation. Because most of leptospirosis patients came to hospital on various day of fever, so it was hard to test biomarkers only on the first day of fever. Third, we have tested NGAL in plasma in addition to urine. This will solve the limitations of unavailable urine sample which is a common situation in severe sepsis/septic shock. Moreover some interventions such as diuretic therapy and intravenous fluid might interfere with the level of urine biomarkers.

In summary, uNGAL and pNGAL appears to be useful markers for detecting AKI in patients with suspected leptospirosis. Our data suggest that neither uNGAL nor pNGAL improves clinical risk prediction of renal non-recovery.

## Supporting Information

S1 Table Stepwise analysis for prediction of failure to recover renal function. (DOCX)
