# Supplementary material for: Neutrophil Gelatinase Associated Lipocalin (NGAL) in Leptospirosis Acute Kidney Injury: A Multicenter Study in Thailand
Source: PLoS One. 2015 Dec 2;10(12):e0143367. doi: 10.1371/journal.pone.0143367 (PMC4667882; doi:10.1371/journal.pone.0143367)
Supplement: S1 Table — (DOCX) [file pone.0143367.s001.docx]

**Supplement**

**S1 Table. Stepwise analysis for prediction of failure to recover renal function**

| Model | AUC (95% CI) | |
| --- | --- | --- |
|  | All cases | Leptospirosis cases |
| uNGAL | 0.53 (0.33,0.75) | 0.57 (0.22,0.89) |
| pNGAL | 0.49 (0.27,0.75) | 0.51 (0.18,0.85) |
| BT | 0.44 (0.26,0.62) | 0.45 (0.15,0.75) |
| SBP | 0.41 (0.23,0.58) | 0.30 (0.04,0.57) |
| uNGAL + pNGAL | 0.55 (0.26,0.78) | 0.59 (0.23,0.95) |
| uNGAL + pNGAL + BT | 0.69 (0.46,0.92) | 0.58 (0.23,0.93) |
| uNGAL + pNGAL + SBP | 0.74 (0.54,0.94) | 0.74 (0.22,1.00) |
| uNGAL + pNGAL + BT + SBP | 0.80 (0.44,1.00) | 0.77 (0,1.00) |

BT: body temperature, SBP: systolic blood pressure
